# Phosphonium-based disinfectants are as effective as best-in-class quaternary ammonium compounds in the eradication of established biofilms in antibiotic-resistant bacteria

**DOI:** 10.1128/spectrum.00556-26

**Published:** 2026-08-04

**Authors:** Elise L. Bezold, Caroline M. Casey, Diana McDonough, Kevin P. C. Minbiole, William M. Wuest

**Affiliations:** 1 Department of Chemistry, Emory University 221304, Atlanta, Georgia, USA; 2 Department of Chemistry and Biochemistry, Villanova University 8210, Villanova, Pennsylvania, USA; The Pennsylvania State University, University Park, Pennsylvania, USA

**Keywords:** biofilm eradication, disinfectants, QAC, QPC, *Staphylococcus aureus*

## Abstract

Disinfectants play a crucial role in infection prevention and control, acting as our first line of defense against pathogenic bacteria. Despite biofilms being a major contributor to recalcitrant infections, the development of novel disinfectants often focuses on bacterial growth inhibition. To address this gap, we compared the ability of a structurally diverse set of quaternary ammonium compounds (QACs) and quaternary phosphonium compounds (QPCs) to eradicate biofilms produced by high-priority pathogens: *Staphylococcus aureus* (MSSA and MRSA), *Acinetobacter baumannii*, and *Pseudomonas aeruginosa*. Our findings reveal comparable activity between structurally analogous QACs and QPCs against the panel of five bacteria. Notably, three compounds exhibited improved efficacy against methicillin-susceptible *S. aureus* (MSSA), community- and hospital-acquired methicillin-resistant *S. aureus* (MRSA), and *A. baumannii* (4 to 63 µM) compared with commercially available, benzethonium chloride (BEC), and didecyldimethylammonium chloride (DDAC). Taken together, these results illustrate the need to understand the efficacy of commercial disinfectants in their ability to eradicate biofilms produced by high-priority pathogens to ensure that proper disinfection protocols are designed to prevent the spread of biofilms from high-contact surfaces to patients.

**IMPORTANCE:**

Bacterial biofilms pose a significant threat to human health as many antibiotics are ineffective against this phenotype, rendering biofilm infections difficult to treat. Disinfectants play a critical role in infection prevention; however, their efficacy in eradicating biofilms is often underexplored. In this study, we evaluated a panel of structurally analogous quaternary ammonium compounds (QACs) and quaternary phosphonium compounds (QPCs) for their ability to eradicate biofilms formed by high-priority pathogens. We found that several QACs and QPCs were more effective than commercially available disinfectants, benzethonium chloride (BEC), and didecyldimethylammonium chloride (DDAC) against methicillin-susceptible *S. aureus* (MSSA), methicillin-resistant *S. aureus* (MRSA), and *A. baumannii*. These findings highlight the importance of evaluating the ability of disinfectants to eradicate biofilms to ensure proper disinfection strategies are designed to prevent biofilm-associated infections.

## INTRODUCTION

**Fig 1:**
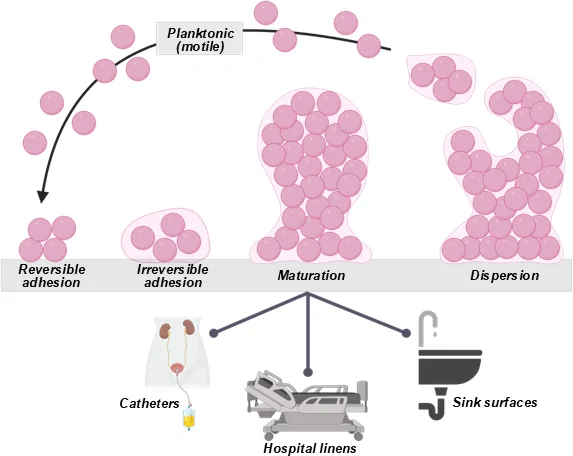
Biofilm life cycle occurs in four stages, with potential places biofilms may be found in the hospital setting. Created in BioRender. Lab, W. (2026) https://BioRender.com/hd5a6ti.

Bacterial communities play vital roles in both health and disease. Globally, bacterial infections are responsible for nearly 14% of deaths each year, with approximately three million infections caused by antibiotic-resistant pathogens (1). However, about 80% of chronic infections are caused by bacterial biofilms (2). Biofilms are complex, structured communities of bacteria encased in a protective extracellular matrix composed of polysaccharides, proteins, and extracellular DNA (3). Biofilm phenotypes also contribute significantly to microbial tolerance to traditional drugs and multidrug resistance (4). This increased tolerance is most notably due to the biofilm matrix, which limits the penetration of many antimicrobials, making biofilms exceptionally difficult to treat and eradicate (5, 6). The biofilm life cycle occurs in four stages (Fig. 1): (i) reversible adhesion, where the planktonic (motile) cells attach to a surface via their pole or flagella, followed by (ii) irreversible adhesion, where the production of the biofilm matrix begins and the cells show increased drug tolerance; (iii) maturation, where cell clusters and microcolonies appear, ranging from 10 µm to 100 µm thick; and finally, (iv) dispersion, where degradation of matrix components begins and cells become motile again, thus showing increased drug susceptibility (7–9). Bacterial biofilms are found in a plethora of places in hospitals, such as sink drains and surfaces, to medical implants like heart valves and catheters, to hospital linens, and biofilms can further be transferred to patients, leading to recalcitrant infections (10, 11). One key way to prevent biofilm infections is via proper sanitation of high-contact surfaces through disinfectants.

Disinfectants serve as critical tools in infection prevention and control, acting as a frontline defense against pathogenic bacteria in both clinical and domestic settings. One of the most widely used classes of disinfectants are cationic biocides, which include subclasses such as biguanides and quaternary ammonium compounds (QACs), like benzethonium chloride (BEC) and didecyldimethyl ammonium chloride (DDAC) (12). However, resistance to QACs has been on the rise since the COVID-19 pandemic, where disinfectant usage increased drastically (13). Therefore, there has been an increased push to diversify the chemical space surrounding cationic biocides, particularly through quaternary phosphonium compounds. Certain QPCs have exhibited greater potency compared with their QAC counterparts in terms of growth inhibition, which could be due to the differences in electronic characteristics (14–18).

**Fig 2:**
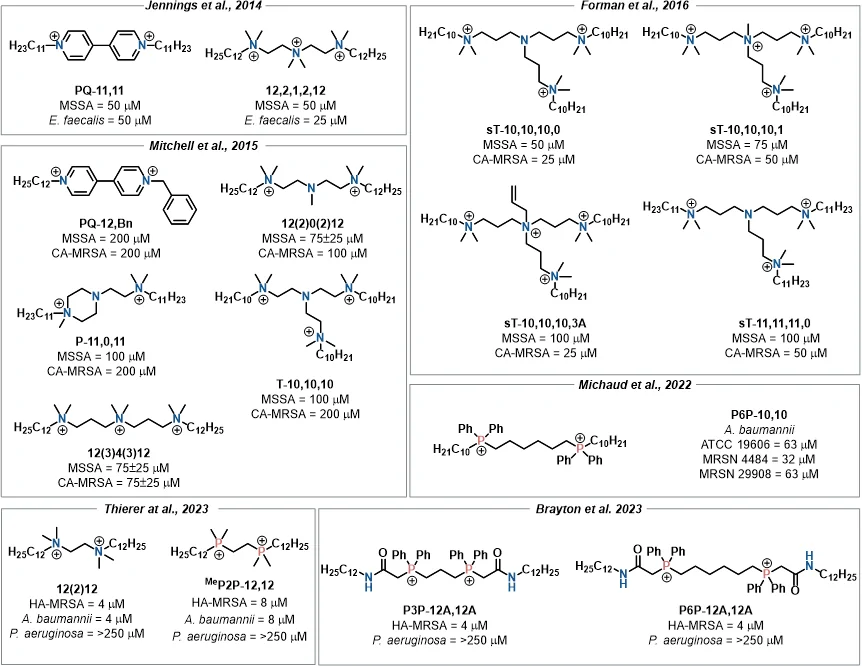
Previous work on the biofilm eradication efficacy for novel disinfectant compounds. Strain and MBEC values indicated.

Expanding past growth inhibition, there have been very few recent reports surrounding the ability of QACs and QPCs to eradicate pre-established biofilms. Ambrosino et al. tested formulations of BAC and DDAC against preformed *Staphylococcus aureus* and *Escherichia coli* biofilms and found that 76% of the formulations tested exhibited 40% eradication (19). Jennings et al. reported a panel of antimicrobial peptide QAC mimics exhibiting a range of biofilm eradication activity against methicillin-susceptible *S. aureus* (MSSA) and *Enterococcus faecalis* with their best-in-class QACs showing an MBEC as low as 50 and 25 µM against MSSA and *E. faecalis* (20). Mitchell et al. reported four novel QAC scaffolds with better eradication activity compared with both BAC (>200 µM for both MSSA and CA-MRSA) and DDAC (MSSA = 150 ± 50 µM and ≥200 µM for CA-MRSA) against MSSA and MRSA biofilms (21). In 2016, Forman *et al*. investigated the role of cationic charge in biological activity, reporting 24 “superT” QACs with three to four different quaternary nitrogen centers. The authors reported only four compounds that outperformed BAC in eradicating both MSSA and CA-MRSA biofilms, with some reported scaffolds being selective against CA-MRSA biofilms. Additionally, they found that the number of quaternary centers had no effect on anti-biofilm activity (22). A summary of these studies is shown in Fig. 2.

The literature surrounding the ability of QPCs to eradicate pre-established biofilms is rather sparse. Many studies have focused on the ability of QPCs to inhibit biofilm formation in a variety of gram-positive and -negative bacteria, with QPCs showing effective inhibition of gram-positive biofilms compared with gram-negative biofilms (23–28). In 2022, Michaud et al. reported that a novel QPC, P6P-10,10, exhibited greater eradication activity against two extensively drug-resistant strains of *Acinetobacter baumannii*, where BAC (500 µM) and DDAC (63 to 125 µM) showed modest activity against the strains (Fig. 2) (16). Thierer et al. showed that QPCs bearing two methyl groups rather than two phenyl groups at the quaternary center exhibited superior eradication activity compared with DDAC when tested against HA-MRSA, *E. faecalis*, and *A. baumannii* biofilms; however, there was minimal difference in potency observed between the QAC and QPC counterparts (Fig. 2) (18). Furthermore, Brayton et al. investigated the eradication activity of two QPCs bearing amide side chains and observed strong activity in the eradication of hospital-acquired methicillin-resistant *S. aureus* (MRSA) biofilm in a slightly preferable level compared with DDAC (Fig. 2) (29). However, the compounds did not show any activity in their ability to eradicate *P. aeruginosa* biofilms (29). These reports exhibit the potential biofilm eradication advantage that QPCs may have compared with QACs; however, a direct comparison between QAC and QPC structural analogs remains limited.

**Fig 3:**
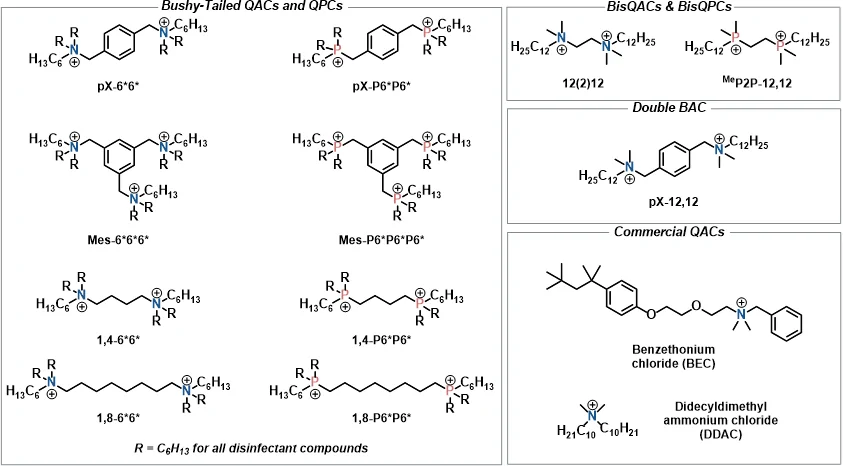
Panel of a structurally diverse set of QAC and QPC structural analogs used in this study, including two commercial QACs, BEC and DDAC.

Therefore, we sought to compare the efficacy of selected QAC and QPC structural analogs that previously exhibited potent growth inhibition activity against pre-established biofilms formed by *S. aureus*, *A. baumannii*, and *P. aeruginosa* (Fig. 3) (17, 18, 30–32). These pathogens are considered high-priority public health threats due to their ability to evade traditional antimicrobials and observed resistance to common therapeutics (33). Furthermore, our interest in comparing QAC and QPC structural analogs was twofold. First, we were interested in whether there would be a biological activity advantage among the QPCs compared with their QAC counterparts, and second, we were interested to see if our novel scaffolds would outperform two well-used disinfectants, BEC and DDAC. From a synthetic perspective, we found that the QAC and QPC structural analogs were readily prepared from trialkylamine and trialkylphosphine subunits, each of which was utilized as nucleophiles to produce compounds differing only in the heteroatom incorporated.

## RESULTS AND DISCUSSION

### Minimum inhibitory concentration

The bioactivities of the QACs and QPCs were determined using minimum inhibitory concentration (MIC) assay via microbroth dilution. Benzethonium chloride (BEC) and didecyldimethylammonium chloride (DDAC) served as control compounds. To determine the MIC values, the compounds were screened against three strains of *S. aureus*: methicillin-susceptible (MSSA), community-acquired (CA-MRSA), and hospital-acquired methicillin-resistant (HA-MRSA). The compounds were also screened against two gram-negative bacteria: *A. baumannii* and *P. aeruginosa*. The concentration required to lyse 20% of red blood cells (Lysis_20_) served as a proxy for cytotoxicity. In Table 1, the QAC and QPC pairs are grouped together, where in each pair, the top compound is the QAC, and the second compound is the QPC.

**TABLE 1:** Biological activity and lysis20 data for QAC and QPC structural analogs compared with commercially available QACs^*a*^

	Minimum inhibitory concentration (µM)					
	MSSA	CA-MRSA	HA-MRSA	*A. baumannii*	*P. aeruginosa*	Lysis_20_
pX-6*6*	0.5	0.5	16	32	32	125
pX-P6*P6*	0.5	0.5	**2**	**8**	**8**	32
Mes-6*6*6*	0.5	1	2	4	8	8
Mes-P6*P6*P6*	1	2	**4**	4	16	8
1,4-6*6*	0.5	0.5	32	125	250	125
1,4-P6*P6*	1	1	**4**	**32**	**32**	63
1,8-6*6*	0.5	0.5	16	125	250	125
1,8-P6*P6*	2	2	4	**16**	**16**	32
12(2)12	1	1	2	2	16	8
MeP2P-12,12	1	1	2	2	8	8
pX-12,12	1	1	2	2	16	8
BEC	2	2	32	32	63	63
DDAC	1	1	4	8	16	8

^*a*^ The QAC is listed first in each pair followed by the QPC. pX-12,12, BEC, and DDAC served as control compounds. Shaded values indicate preferential growth inhibition activity. Bolded values indicate preferential bioactivity compared to QAC structural analog. All values are an average of three independent replicates (raw data can be found in Supplemental material).

Bioactivity for the compound pairs against MSSA and CA-MRSA appeared to be identical, and our reported compounds exhibited bioactivity comparable to the commercially available QACs, BEC and DDAC. However, we did observe bioactivity differences between the QAC and QPC pairs against HA-MRSA, *A. baumannii*, and *P. aeruginosa*. We observed that the QPCs for each pair exhibited improved bioactivity compared with their QAC counterpart. This was particularly noticeable in pX-P6*P6*, 1,4-P6*P6*, and 1,8-P6*P6* where each compound showed a two- to three-fold reduction in MIC against the three strains. Previous reports have demonstrated that structurally similar QPCs to 1,4-P6*P6* and 1,8-P6*P6*, which are categorized as bolaamphiphiles, selectively target the inner membrane of gram-negative bacteria compared with the indiscriminate membrane disruption of QACs, giving rise to the potent bioactivity observed with QPCs (15, 34). We hypothesize this distinct mechanism of action could explain the differences in bioactivity observed in this report. Notably, the QPCs exhibited more potent bioactivity when compared with BEC; however, the bioactivity was comparable to DDAC.

Aside from differing mechanisms of action for QACs and QPCs, it has been shown that the privileged bioactivity observed with QPCs may arise from differences in electronic character, where the phosphonium atom bears a higher cationic charge, aiding in the adsorption of QPCs into the negatively charged bacterial cell membrane (18, 35), thus leading to an increase in antimicrobial activity (18, 35). This has further been rationalized through NMR analysis of 12(2)12 and MeP2P-12,12, where Thierer et al. observed that the methyl protons adjacent to each cationic center exhibited differences in chemical shift (18). Furthermore, we hypothesize that this rationalization can be applied to explaining the differences in bioactivity between the QAC and QPC structural analogs used in this study, as we observed increased bioactivity for the QPCs compared with their QAC structural analogs, most prominently against the gram-negative bacteria, *A. baumannii* and *P. aeruginosa*. In terms of cytotoxicity, we observed that QACs generally displayed lower toxicity compared with their QPC counterparts. With that being said, many QPCs exhibited lysis values that were comparable to DDAC at 8 µM.

### Minimum biofilm eradication concentration (MBEC)

Next, we sought to determine the minimum biofilm eradication concentration for our compounds. Minimum biofilm eradication concentration is the lowest concentration that can eliminate a preformed biofilm (36). Since disinfectants are typically used on high-contact surfaces where biofilm may already inhabit, we were interested in the ability of our compounds to eradicate preestablished biofilms, as theoretically, this would be used as a method to prevent infection.

Generally, we observed that our panel of compounds was effective at eradicating MSSA and CA-MRSA pre-established biofilms, with only a handful of QACs exhibiting poor bioactivity (Table 2). Notably, we observed potent eradication activity with 1,4-P6*P6* against MSSA and CA-MRSA biofilms compared with the QAC counterpart, which showed no activity. We also observed that against preestablished CA-MRSA biofilms, 1,8-P6*P6* (16 µM) exhibited preferential activity compared 1,8-6*6* (63 µM). Importantly, our novel compounds exhibited preferential bioactivity compared with commercially available disinfectants, BEC and DDAC.

**TABLE 2:** Minimum biofilm eradication concentration for QAC and QPC structural analogs compared with commercially available QACs, BEC and DDAC^*a*^

	Minimum biofilm eradication concentration (µM)				
	MSSA	CA-MRSA	HA-MRSA	*A. baumannii*	*P. aeruginosa*
pX-6*6*	8	8	>250	>250	>250
pX-P6*P6*	4	8	125	>250	>250
Mes-6*6*6*	4	8	125	>250	>250
Mes-P6*P6*P6*	8	16	63	125	>250
1,4-6*6*	>250	>250	>250	>250	>250
1,4-P6*P6*	**8**	**8**	>250	>250	>250
1,8-6*6*	16	63	>250	>250	>250
1,8-P6*P6*	16	16	>250	250	250
12(2)12	16	63	**32**	**16**	125
MeP2P-12,12	16	32	**32**	**16**	125
pX-12,12	**4**	32	**32**	**16**	125
BEC	32	63	>250	>250	>250
DDAC	16	32	250	>250	>250

^*a*^ All values are an average of three independent replicates (raw data can be found in Supplemental material). Shaded values indicate preferential biofilm eradication. Bolded values indicate favorable activity compared to structural analogs.

In terms of biofilm eradication against gram-negative bacteria, we were pleased to see that three compounds showed favorable activity against *A. baumannii* biofilms. 12(2)12, MeP2P-12,12, and pX-12,12 showed an MBEC of 16 µM, in stark contrast to BEC and DDAC at >250 µM. Previously, we reported that 12(2)12 and MeP2P-12,12 exhibited activity against *A. baumannii* biofilms at 4 and 8 µM, respectively (18), which differs from our current data. The differences in eradication activity between this report and Thierer *et al*. could arise from variations in growth conditions and experimental conditions. Thierer et al. used tryptic soy broth (TSB) and pegged lids for their biofilm eradication assay (18), whereas we utilized Mueller-Hinton broth (MHB) and surface-treated flat bottom plates. It is important to note that these two experimental conditions are measuring different biofilm phenotypes: pegged lids interrogate pellicle formation at the air-liquid interface and surface-treated plates study biofilm formation at the solid-liquid interface. These conditions can differentially influence biofilm growth, leading to differences in robustness, therefore altering bioactivity of a given disinfectant.

It is not unsurprising that many of the tested compounds were unable to eradicate pre-established biofilm produced by gram-negative *A. baumannii* and *P. aeruginosa* compared with gram-positive *S. aureus*. One possible hypothesis for this spectrum of activity could be the differences in membrane structure and biofilm matrix composition. Gram-negative bacteria harbor an outer membrane composed of the glycolipid, lipopolysaccharide (LPS), whereas gram-positive bacteria lack this extra membrane (37, 38). The outer membrane plays a key role in protecting gram-negative bacteria from exogenous stressors, often making these bacteria harder to kill and eradicate. Furthermore, biofilm matrices produced by gram-negative bacteria harbor outer membrane vesicles composed of lipoproteins and LPS, which could function to eradicate exogenous materials (38). We did not observe major differences in eradication activity in terms of the number of cationic residues between the pX-6*6*/pX-P6*P6* series and the Mes-6*6*6*/Mes-P6*P6*P6* series. We did observe that the carbon linker between ammonium residues may influence bioactivity, where 1,8-6*6* exhibited a fivefold increase in bioactivity compared with 1-4-6*6* against MSSA and CA-MRSA biofilms; however, this trend was not observed with tested QPCs. This trend differs from Jennings *et al.,* where the authors found that the spatial distribution of charge was not crucial for biofilm eradication activity (20). It is important to note that the tested compounds featured two methyls and a 12-carbon chain, whereas our tested compounds exhibit three six-carbon chains on the ammonium residue, and the number of alkyl chains may have an influence on biofilm eradication activity.

### Assessment of metabolic activity of biofilm cells

At high concentrations for some of the tested compounds, we observed increased crystal violet (CV) staining. CV is a non-specific stain, meaning it does not discriminate between live and dead cells (36). Therefore, we utilized a tetrazolium salt dye, 2,3,5-triphenyl tetrazolium chloride (TTC), to assess metabolically active cells. In the presence of metabolically active cells, TTC can be reduced into the red formazan product, and the absorbance of this compound can be quantified (36). A higher absorbance correlates to metabolically active cells. This phenomenon was not conserved to only QACs or QPCs, nor was it conserved among pairs. The only pair of active compounds that exhibited high CV staining at high compound concentration was 12(2)12 and MeP2P-12,12, while the other compounds that exhibited this phenomenon were Mes-P6*P6*P6* and pX-12,12.

**Fig 4:**
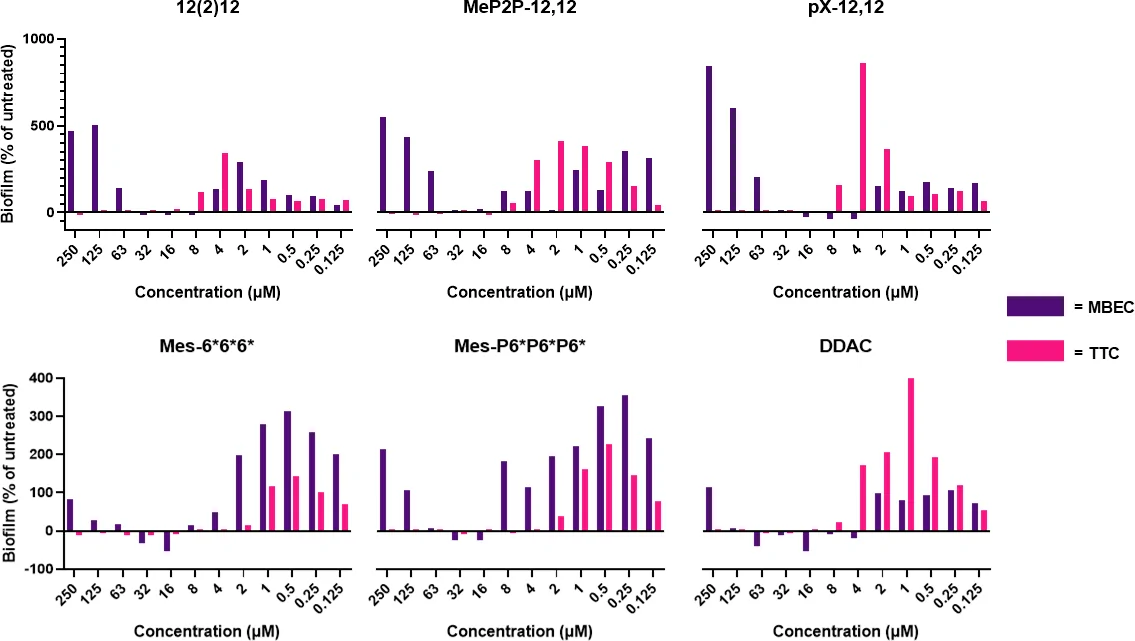
Quantification of crystal violet staining and metabolic activity using TTC dye to assess whether biofilms are metabolically active at high disinfectant concentrations. Purple represents CV staining, and pink represents TTC. Data are the averages of three independent replicates.

We found that the biofilms treated with high concentrations of the compound (63 to 250 µM) were not metabolically active, as shown in the plots below (Fig. 4). We hypothesize that the increased CV staining could reflect cells with damaged membranes. Taken together, these results show the importance of running two assays that assess metabolic activity and biofilm mass in parallel.

### Confocal microscopy

We also utilized confocal laser scanning microscopy to visualize phenotypic responses that QACs and QPCs may elicit in MSSA (SH1000). We chose to study this strain as it produced robust biofilms compared with CA-MRSA and HA-MRSA, which would allow for ease of imaging. In our studies, we used two separate dyes to visualize the biofilm cells: propidium iodide (PI) and SYTO 9. PI stains cells with damaged membranes red, while SYTO 9 stains cells with both intact and damaged membranes green (39). When the PI and SYTO 9 images are merged, we can visualize a reduction in green fluorescence in areas with loss of membrane integrity, which reflects decreased cell viability (39).

**Fig 5:**
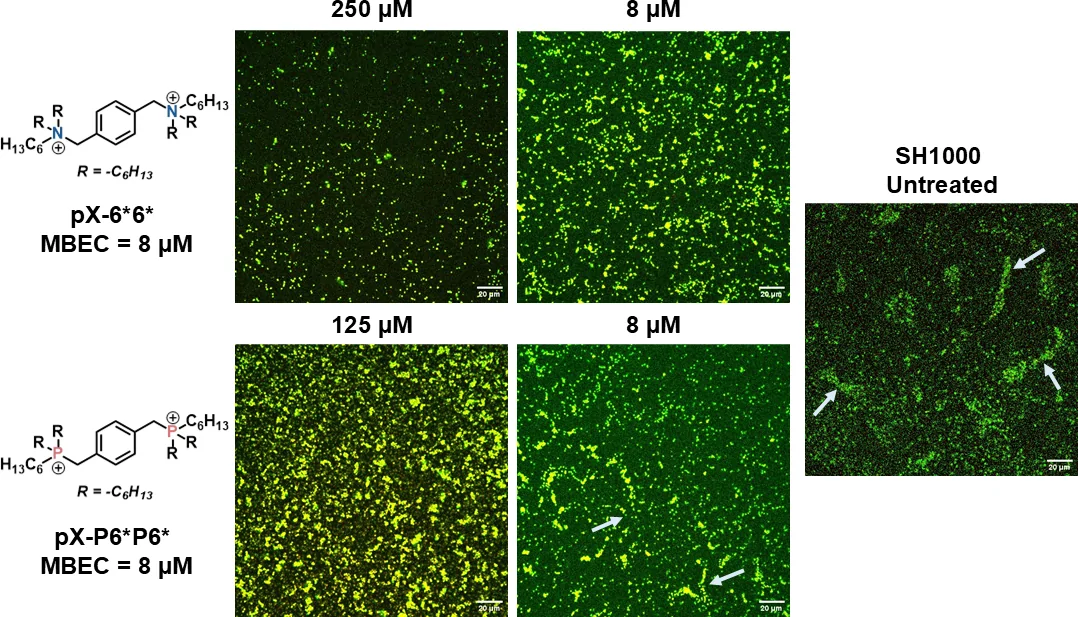
MSSA (SH1000) biofilm response to treatment of disinfectant compounds. Top images show biofilm treated with pX-6*6*. Bottom images show biofilm treated with pX-P6*P6*. Confocal images were taken with a 60× objective and LIVE/DEAD stain was used to visualize cell viability. Images contain a 20-µm scale bar. Individual channel images are shown in Fig. S1 to S3 in Supplemental material.

As shown in Fig. 5, we observed many biofilm colonies in our untreated MSSA control, which are pointed out by the blue arrows. When the untreated control was compared with the bacteria treated with concentrations of pX-6*6* at 250 and 8 µM (Fig. 5, top), we observed a disruption of the biofilm colonies, with more bacterial density present as MBEC was approached, as well as more single-cell growth at high concentration. We observed a similar phenomenon for MSSA biofilms treated with pX-P6*P6*; however, as MBEC is approached, some biofilm colonies begin to appear, as pointed out by the blue arrows (Fig. 5, bottom). Interestingly, even at high concentrations of pX-P6*P6*, we observed larger bacterial density than with pX-6*6*. When these qualitative results are taken together, we conclude that QACs may be more efficient in disrupting biofilm formation than the QPC counterpart.

However, caution should be taken with this conclusion, as it can be challenging to prepare uniform biofilms, given the laborious nature of the protocol, where many factors can disrupt the adhered biofilms, such as removing planktonic cells via inversion of the plate or aspiration with a micropipette.

### Conclusions

Biofilms are complex, structured communities protected by an extracellular matrix. This matrix contributes significantly to the increased tolerance observed toward antimicrobials, as many antimicrobials cannot penetrate this matrix. Disinfectants are a key tool in preventing infections, especially in hospitals, where biofilms colonize many surfaces and can be transferred to patients, leading to harder-to-treat infections. Therefore, there is a significant need to understand and discover novel disinfectant compounds that can eradicate biofilms in high-priority pathogens.

We sought to investigate how well our novel disinfectant compounds could eradicate biofilm produced by MSSA, CA-MRSA, HA-MRSA, *A. baumannii*, and *P. aeruginosa*. We hypothesized that our novel QPCs may show enhanced biofilm eradication capabilities compared with the QAC counterpart. We found that, generally, our QACs and QPCs exhibited similar biofilm eradication abilities against MSSA and CA-MRSA, but overall, our novel disinfectant compounds exhibited notably better bioactivity than BEC and DDAC. Though our panel of compounds showed low activity against HA-MRSA and *A. baumannii* biofilm, three compounds did exhibit an MBEC value of 16 or 32 µM: 12(2)12, MeP2P-12,12, and pX-12,12. Furthermore, confocal microscopy showed that our compounds were effective in disrupting biofilm in MSSA; however, our images do show that the QACs may be more efficient in disrupting biofilm formation compared with QPCs. In conclusion, this study underscores the necessity of understanding the efficacy of novel QACs and QPCs in their abilities to eradicate biofilm produced by high-priority pathogens, as this can inform proper disinfection protocols to prevent biofilm infections in immunocompromised patients.

## MATERIALS AND METHODS

### Biological assays

For all biological assays, laboratory strains of methicillin-susceptible *Staphylococcus aureus* MSSA (SH1000), community-acquired methicillin-resistant *Staphylococcus aureus* (USA300-0114), hospital-acquired methicillin-resistant *Staphylococcus aureus* (ATCC 33591), *Pseudomonas aeruginosa* (PAO1), and *Acinetobacter baumannii* (ATCC 17978) were streaked onto LB agar plates from freezer stocks and incubated statically for 18 h at 37°C. Single colonies were used to inoculate 5 mL of the indicated media: SH1000, USA300-0114, PAO1, and ATCC 17978 were grown in Difco cation-adjusted Mueller-Hinton (MHB) broth, whereas ATCC 33591 was grown in Bacto tryptic soy broth (TSB). Cultures were grown at 37°C for 18 h with shaking (200 rpm). Optical density (OD) measurements were obtained using a BioTek Synergy H1 Hybrid plate reader (Santa Clara, CA, USA).

### Compound synthesis

The QAC and QPC compounds used herein were prepared according to literature methods (17, 18, 30–32, 40), with the exception of 1,8-6*6* whose synthesis is described here. To a 20-mL reaction vial with a stir bar and a pressure-relieving septum cap were added 1,8-dibromooctane (181 µL, 0.272 g, 1.00 mmol), acetonitrile (10 mL), isopropanol (0.5 mL), and trihexylamine (848 µL, 0.674 g, 2.50 mmol). The reaction vial was placed into a reaction pie block preheated on a stir plate to 65°C for 4 days. After cooling to room temperature, the reaction mixture was concentrated *in vacuo*. The product was purified by column chromatography (silica, 1:19 methanol: dichloromethane) to afford 1,8-6*6* as a pale-yellow oil (0.0620 g, 8%). ^1^H NMR (400 MHz, CDCl_3_): δ 3.38–3.26 (m, 16H), 1.79 (p, *J* = 6.9 Hz, 4H), 1.70–1.59 (m, 12H), 1.37–1.19 (m, 44H), 0.88–0.80 (m, 18H). ^13^C{^1^H} NMR (101 MHz, CDCl_3_): δ 59.3, 34.1, 32.6, 31.3, 29.0, 28.5, 27.9, 26.3, 26.1, 22.5, 22.4, 14.0. HRMS (ESI+): Found 325.3701, C_44_H_94_N_2_ [M-2Br^-^]^2+^ requires 325.3703 m/z.

### Minimum inhibitory concentration (MIC)

The compounds were prepared in a 1.0 mM, 9:1 water: DMSO solution. To determine the MIC values, compounds were serially diluted with sterile deionized water twofold from stock solutions (1.0 mM) to yield 12 100-μL test concentrations (250 to 0.125 μM) wherein the starting concentration of dimethyl sulfoxide (DMSO) was 2.5%. Overnight bacterial cultures were regrown from a 1:100 dilution in fresh MHB or TSB until mid-exponential phase was achieved as determined by the optical density recorded at 600 nm (OD_600_). All cultures were then diluted again to ca. 10^6^ CFU/mL, and 100 μL was inoculated into each well of a U-bottom 96-well plate (VWR, 10861-564) containing 100 μL of compound solution. Plates were incubated statically at 37°C for 72 h upon which wells were evaluated visually for bacterial growth. The MIC was determined as the lowest concentration of compound resulting in no bacterial growth visible to the naked eye based on the average in three independent experiments. Media (MHB or TSB), aqueous 10% DMSO (Millipore Sigma, MX1458-6), and bacterial (no treatment) controls were conducted for each strain. Standard deviations were calculated using the “ST.DEV.S” function on Microsoft Excel for Microsoft 365.

### Red blood cell (RBC) lysis assay (Lysis_20_)

RBC lysis assay was performed following the procedure described by Peng et al. with minor modification (41). RBC lysis assays were performed on mechanically defibrinated sheep blood (Hemostat Labs, DSB030). An aliquot of 1.5-mL blood was placed into a microcentrifuge tube and centrifuged at 10,000 rpm for 10 min. The supernatant was removed, and the cells were resuspended with 1 mL of phosphate-buffered saline (PBS). The suspension was centrifuged as described above, the supernatant was removed, and cells were resuspended four additional times in 1 mL PBS. The final cell suspension was diluted 20-fold with PBS. Compounds were serially diluted with PBS twofold from stock solutions (1.0 mM, 9:1 water: DMSO) to yield 100 µL of 12 test concentrations (250 to 0.125 μM) on U-bottom 96-well plate (VWR, 10861-564), wherein the starting concentration of DMSO was 2.5%. To each of the wells, 100 µL of the 20-fold suspension dilution was then inoculated. The concentration of DMSO in the first well was 2.5%, resulting in DMSO-induced lysis at all concentrations >63 µM. TritonX (1% by volume) served as a positive control (100% lysis marker) and sterile PBS served as a negative control (0% lysis marker). Samples were then placed in an incubator at 37°C and shaken at 200 rpm. After 1 h, the samples were centrifuged at 2,000 rpm for 10 min. Then, 100 μL of supernatant was then transferred to a fresh flat-bottom 96-well plate (Falcon, 351172), and the absorbance of the supernatant was measured with a UV spectrometer at a 540 nm wavelength. The concentration inducing 20% RBC lysis was then calculated for each compound based upon the absorbances of the 1% TritonX and PBS controls. The data were averaged across three replicates. Aqueous DMSO controls were conducted as appropriate for each compound.

### Minimum biofilm eradication concentration determination (MBEC)

Minimum biofilm eradication concentration was performed following the procedure described by Brayton et al. and Yang et al. with minor modification (29, 42). Overnight bacterial cultures were regrown from a 1:100 dilution in fresh MHB or TSB media until mid-exponential phase was achieved as determined by the optical density recorded at 600 nm (OD_600_). All cultures were diluted again to ca. 10^6^ CFU/mL, and 125 μL of bacterial culture was inoculated into each well of a surface-treated flat bottom 96-well plate (Falcon, 353072). Plates were incubated statically at 37°C for 24 h to establish biofilms.

After 24 h, planktonic growth was removed via aspiration and plates were washed thrice with 200 μL sterile PBS. To prepare the “challenge plate” (plate containing serial dilutions of compounds), compounds were serially diluted with sterile deionized water twofold from stock solutions (1.0 mM, 9:1 water: DMSO) to yield twelve 100-μL test concentrations (250 to 0.125 μM) on a U-bottom 96-well plate (VWR, 10861-564), wherein the starting concentration of dimethyl sulfoxide (DMSO) was 2.5%. An additional 100 μL of media was added, and then the test concentrations were transferred to the flat-bottom plate containing the pre-established biofilms. The plates were incubated statically at 37°C for 24 h. Then, the spent media were removed from each well, biofilms were washed thrice with 200 μL sterile PBS, and 200 μL of fresh media was added. The plates were incubated statically at 37°C for 24 h. The biofilms were then visually inspected, and the MBEC was determined as the lowest test concentration that resulted in the eradicate biofilm (i.e. wells displaying no turbidity). The data were averaged across three replicates. Standard deviations were calculated using the “ST.DEV.S” function on Microsoft Excel for Microsoft 365.

### Crystal violet biofilm quantification assay

Crystal violet staining assay was performed following the procedure described by Haney et al. with minor modification (36). Overnight bacterial cultures were regrown from a 1:100 dilution in fresh MHB or TSB media until mid-exponential phase was achieved as determined by the optical density recorded at 600 nm (OD_600_). All cultures were diluted again to ca. 10^6^ CFU/mL, and 125 μL of bacterial culture was inoculated into each well of a surface-treated flat bottom 96-well plate (Falcon, 353072). Plates were incubated statically at 37°C for 24 h to establish biofilms.

After 24 h, planktonic growth was removed via aspiration, and plates were washed thrice with 200 μL sterile PBS. To prepare the “challenge plate” (plate containing serial dilutions of compounds), compounds were serially diluted with sterile deionized water twofold from stock solutions (1.0 mM, 9:1 water: DMSO) to yield 12 100-μL test concentrations (250 to 0.125 μM) on a U-bottom 96-well plate (VWR, 10861-564), wherein the starting concentration of dimethyl sulfoxide (DMSO) was 2.5%. An additional 100 μL of media was added, and then the test concentrations were transferred to the flat-bottom plate containing the pre-established biofilms. The plates were incubated statically at 37°C for 24 h.

After incubation, the spent growth media were removed via aspiration, carefully so as not to disturb the adhered biofilm, and plates were washed thrice with 200 μL sterile PBS. The plates were then inverted on a paper towel and gently tapped to remove excess liquid. The biofilm in each well was stained with 200 μL of 0.1% (wt/vol) crystal violet (CV) solution. The plates were stained for 30 min at room temperature with shaking (100 rpm). After incubation, the CV staining solution was discarded, and wells were rinsed three times with deionized water. After washing, the plates were inverted and tapped on a paper towel to remove excess liquid. CV was then solubilized with 200 μL of 70% ethanol and incubated for 30 min at room temperature with shaking (100 rpm). Then, 100 μL of solubilized CV was transferred to a fresh flat-bottom 96-well plate (Falcon, 351172), and the A_595_ was recorded using a BioTek Synergy H1 Hybrid plate reader (Santa Clara, CA). The data were averaged across three replicates.

The percentage biofilm growth was calculated with the following equation:


$$\%\;biofilm\;growth=\frac{\left(Sample_{A595}-SC_{A595}\right)}{GC_{A595}-SC_{A595}}\times 100$$

where SC_A595_ = sterility control (0% bacterial growth)

GC_A595_ = growth control (100% bacterial growth)

### TTC assay

Tetrazolium dye assay was performed following the procedure described by Haney et al. with minor modification (36). Overnight bacterial cultures were regrown from a 1:100 dilution in fresh MHB or TSB media until mid-exponential phase was achieved as determined by the optical density recorded at 600 nm (OD_600_). All cultures were diluted again to ca. 10^6^ CFU/mL, and 125 μL of bacterial culture was inoculated into each well of a surface-treated flat bottom 96-well plate (Falcon, 353072). Plates were incubated statically at 37°C for 24 h to establish biofilms.

After 24 h, planktonic growth was removed via aspiration, and plates were washed thrice with 200 μL sterile PBS. To prepare the “challenge plate” (plate containing serial dilutions of compounds), compounds were serially diluted with sterile deionized water twofold from stock solutions (1.0 mM, 9:1 water: DMSO) to yield 12 90-μL test concentrations (250 to 0.125 μM) on a U-bottom 96-well plate (VWR, 10861-564), wherein the starting concentration of dimethyl sulfoxide (DMSO) was 2.5%. An additional 90 μL of media was added, and then the test concentrations were transferred to the flat-bottom plate containing the pre-established biofilms. Then, 2 μL of a 5% (wt/vol) sterile-filtered 2,3,5-triphenyl tetrazolium chloride (TTC) in deionized water solution was added to all test concentrations, including DMSO, bacteria, and media controls. The final concentration of TTC in each well was 0.05% (wt/vol). The plates were incubated statically at 37°C for 24 h.

After incubation, the spent growth media were removed via aspiration, carefully so as not to disturb the adhered biofilm, and plates were washed thrice with 200 μL sterile PBS. The plates were then inverted on a paper towel and gently tapped to remove excess liquid. The TTC dye was solubilized with 200 µL of DMSO and incubated for 30 min at room temperature with shaking (100 rpm). Then, 100 μL of solubilized TTC dye was transferred to a fresh flat-bottom 96-well plate (Falcon, 351172), and the A_500_ was recorded using a BioTek Synergy H1 Hybrid plate reader (Santa Clara, CA). The data were averaged across three replicates.

The percentage biofilm metabolism was calculated with the following equation:


$$\%\;biofilm\;metabolism=\frac{\left(Sample_{A500}-SC_{A500}\right)}{GC_{A500}-SC_{A500}}\times 100$$

where SC_A500_ = sterility control (0% bacterial growth)

GC_A500_ = growth control (100% bacterial growth).

### Confocal microscopy

Overnight bacterial cultures were regrown from a 1:100 dilution in fresh MHB or TSB media until mid-exponential phase was achieved as determined by the optical density recorded at 600 nm (OD_600_). All cultures were diluted again to ca. 10^6^ CFU/mL, and 100 μL of bacterial culture was inoculated into each well of an uncoated 96-well plate with 5-mm glass diameter from MatTek (Part No: P96G-1.5-5-F). Plates were incubated statically at 37°C for 24 h to establish biofilms.

After 24 h, planktonic growth was removed via aspiration, and plates were washed thrice with 200 μL sterile PBS. To prepare the “challenge plate” (plate containing serial dilutions of compounds), compounds were serially diluted with sterile deionized water twofold from stock solutions (1.0 mM, 9:1 water: DMSO) to yield 12 100-μL test concentrations (250 to 0.125 μM), where in the starting concentration of dimethyl sulfoxide (DMSO) was 2.5%. An additional 100 μL of media was added and then the test concentrations were transferred to the glass flat-bottom plate containing the pre-established biofilms. The plates were incubated statically at 37°C for 24 h.

After incubation, the spent growth media were removed via aspiration, carefully as to not disturb the adhered biofilm, and plates were washed thrice with 200 μL sterile deionized water. The plates were then inverted on a paper towel and allowed to dry for 1 h prior to imaging. Then, 20 μL of BacLight LIVE/DEAD stain (Thermo Fisher Scientific, L-7007) was added to each well to be imaged and incubated in the dark for 15 min. Images of biofilms were obtained using a Nikon C2Si confocal microscope with a continuous laser source at 60× oil objective at 488 and 647 nm. Images were processed using Fiji software (43).

## Data Availability

The data supporting the findings of this study are available in the Supplemental material of this article.

## SUPPLEMENTAL MATERIAL

Supplemental tables and figures spectrum.00556-26-s0001.pdf Tables S1 to S10 and Figures S1 to S4.

ASM does not own the copyrights to Supplemental Material that may be linked to, or accessed through, an article. The authors have granted ASM a non-exclusive, world-wide license to publish the Supplemental Material files. Please contact the corresponding author directly for reuse.
