# Supplementary material for: Phosphonium-based disinfectants are as effective as best-in-class quaternary ammonium compounds in the eradication of established biofilms in antibiotic-resistant bacteria
Source: Microbiol Spectr. 2026 Aug 4;14(9):e00556-26. doi: 10.1128/spectrum.00556-26 (PMC13532204; doi:10.1128/spectrum.00556-26)
Supplement: Supplementary file 1 [file spectrum.00556-26-s0001.pdf]

Supplementary Information

Comparative biofilm eradication analysis of structurally analogous QAC and QPC Disinfectants Reveals Minimal Class-Dependent Differences

Elise L. Bezold[a], Caroline M. Casey[b], Diana McDonough[b], Kevin P.C. Minbiole[b]*, and William M. Wuest[a]*

[a]Department of Chemistry, Emory University, Atlanta, GA 30322

[b]Department of Chemistry and Biochemistry, Villanova University, Villanova, PA 19085

Table of Contents

1. **Supporting Tables** **S2-S7**
   Table S1: Raw MIC Data for MSSA (SH100) across three replicates.
   Table S2: Raw MIC Data for CA-MRSA (USA300-0114) across three replicates.
   Table S3: Raw MIC Data for HA-MRSA (ATCC 33591) across three replicates.
   Table S4: Raw MIC Data for *A. baumannii* (ATCC 17978) across three replicates.
   Table S5: Raw MIC Data for *P. aeruginosa* (PAO1) across three replicates.
   Table S6: Raw $Lysis_{20}$ Data across three replicates.
   Table S7: Raw MBEC Data for MSSA (SH1000) across three replicates.
   Table S8: Raw MBEC Data for CA-MRSA (USA300-0114) across three replicates.
   Table S9: Raw MBEC Data for HA-MRSA (ATCC 33591) across three replicates.
   Table S10: Raw MBEC Data for *A. baumannii* (ATCC 17978) across three replicates.
   Table S11: Raw MBEC Data for *P. aeruginosa* (PAO1) across three replicates.
2. **Supporting Figures** **S8-S9**
   Figure S1: Individual channel images for MSSA (SH1000) biofilm response to treatment with **pX-6*6***.
   Figure S2: Individual channel images for MSSA (SH1000) biofilm response to treatment with **pX-P6*P6***.
   Figure S3: Individual channel images for untreated MSSA (SH1000) biofilm.
   Figure S4: Quantification of crystal violet staining and metabolic activity using TTC dye for all compounds.
3. **Spectra for 1,8-6*6*** **S10**

1. Supporting Tables

Table S1. Raw MIC Data for MSSA (SH1000) across three replicates.

	**Concentration (µM)**			
	**Trial 1**	**Trial 2**	**Trial 3**	**Average (±SD)**
**pX-6*6***	0.5	0.5	0.5	**0.5 (±0)**
**pX-P6*P6***	0.5	0.5	0.5	**0.5 (±0)**
**Mes-6*6*6***	0.5	0.5	1	**0.5 (±0.25)**
**Mes-P6*P6*P6***	1	2	1	**1 (±0.5)**
**1,4-6*6***	0.5	0.5	0.5	**0.5 (±0)**
**1,4-P6*P6***	1	1	1	**1 (±0)**
**1,8-6*6***	0.5	0.5	0.5	**0.5 (±0)**
**1,8-P6*P6***	2	1	2	**2 (±0.6)**
**12(2)12**	1	1	1	**1 (±0)**
**MeP2P-12,12**	1	1	2	**1 (±0.5)**
**pX-12,12**	1	1	1	**1 (±0)**
**BEC**	2	2	2	**2 (±0)**
**DDAC**	1	0.5	1	**1 (±0.25)**

Table S2. Raw MIC Data for CA-MRSA (USA300-0114) across three replicates.

	**Concentration (µM)**			
	**Trial 1**	**Trial 2**	**Trial 3**	**Average (±SD)**
**pX-6*6***	1	0.5	0.5	**0.5 (±0.3)**
**pX-P6*P6***	0.5	0.5	0.5	**0.5 (±0)**
**Mes-6*6*6***	1	1	1	**1 (±0)**
**Mes-P6*P6*P6***	2	2	2	**2 (±0)**
**1,4-6*6***	0.5	1	0.5	**0.5 (±0.3)**
**1,4-P6*P6***	1	1	1	**1 (±0)**
**1,8-6*6***	1	0.5	0.5	**0.5 (±0.3)**
**1,8-P6*P6***	2	2	2	**2 (±0)**
**12(2)12**	1	2	1	**1 (±0.6)**
**MeP2P-12,12**	1	1	1	**1 (±0)**
**pX-12,12**	2	1	1	**1 (±0.6)**
**BEC**	1	2	2	**2 (±0.6)**
**DDAC**	1	0.5	1	**1 (±0.3)**

Table S3. Raw MIC Data for HA-MRSA (ATCC 33591) across three replicates.

	**Concentration (µM)**			
	**Trial 1**	**Trial 2**	**Trial 3**	**Average (±SD)**
**pX-6*6***	16	16	16	**16 (±0)**
**pX-P6*P6***	2	2	2	**2 (±0)**
**Mes-6*6*6***	2	2	2	**2 (±0)**
**Mes-P6*P6*P6***	4	2	4	**4 (±1)**
**1,4-6*6***	32	16	32	**32 (±9)**
**1,4-P6*P6***	4	4	8	**4 (±2)**
**1,8-6*6***	32	16	16	**16 (±9)**
**1,8-P6*P6***	8	4	4	**4 (±2)**
**12(2)12**	2	2	4	**2 (±1)**
**MeP2P-12,12**	2	2	2	**2 (±0)**
**pX-12,12**	2	2	2	**2 (±0)**
**BEC**	32	32	32	**32 (±0)**
**DDAC**	4	4	4	**4 (±0)**

Table S4. Raw MIC Data for *A. baumannii* (ATCC 17978) across three replicates.

	**Concentration (µM)**			
	**Trial 1**	**Trial 2**	**Trial 3**	**Average (±SD)**
**pX-6*6***	32	32	32	**32 (±0)**
**pX-P6*P6***	8	8	8	**8 (±0)**
**Mes-6*6*6***	4	4	4	**4 (±0)**
**Mes-P6*P6*P6***	4	4	4	**4 (±0)**
**1,4-6*6***	125	125	125	**125 (±0)**
**1,4-P6*P6***	32	32	32	**32 (±0)**
**1,8-6*6***	125	125	125	**125 (±0)**
**1,8-P6*P6***	32	16	16	**16 (±9)**
**12(2)12**	2	2	2	**2 (±0)**
**MeP2P-12,12**	2	2	2	**2 (±0)**
**pX-12,12**	2	2	2	**2 (±0)**
**BEC**	32	16	32	**32 (±9)**
**DDAC**	8	8	8	**8 (±0)**

Table S5. Raw MIC Data for *P. aeruginosa* (PAO1) across three replicates.

	Concentration (μM)			
	**Trial 1**	**Trial 2**	**Trial 3**	**Average (±SD)**
**pX-6*6***	32	32	32	**32 (±0)**
**pX-P6*P6***	8	8	8	**8 (±0)**
**Mes-6*6*6***	4	8	8	**8 (±2)**
**Mes-P6*P6*P6***	16	16	8	**16 (±5)**
**1,4-6*6***	250	250	250	**250 (±0)**
**1,4-P6*P6***	63	32	32	**32 (±18)**
**1,8-6*6***	250	250	250	**250 (±0)**
**1,8-P6*P6***	16	16	8	**16 (±5)**
**12(2)12**	16	16	4	**16 (±7)**
**MeP2P-12,12**	8	8	16	**8 (±5)**
**pX-12,12**	16	16	16	**16 (±0)**
**BEC**	125	63	63	**63 (±36)**
**DDAC**	16	32	16	**16 (±9)**

Table S6. Raw $Lysis_{20}$ Data across three replicates.

	Concentration (μM)			
	**Trial 1**	**Trial 2**	**Trial 3**	**Average (±SD)**
**pX-6*6***	32	125	125	**125 (±54)**
**pX-P6*P6***	32	63	32	**32 (±18)**
**Mes-6*6*6***	8	8	8	**8 (±0)**
**Mes-P6*P6*P6***	8	8	8	**8 (±0)**
**1,4-6*6***	250	>250	>250	**>250**
**1,4-P6*P6***	63	125	63	**63 (±36)**
**1,8-6*6***	63	63	63	**63 (±0)**
**1,8-P6*P6***	32	32	32	**32 (±0)**
**12(2)12**	8	8	8	**8 (±0)**
**MeP2P-12,12**	8	8	8	**8 (±0)**
**pX-12,12**	8	8	8	**8 (±0)**
**BEC**	63	63	63	**63 (±0)**
**DDAC**	32	16	16	**16 (±9)**

Table S7. Raw MBEC Data for MSSA (SH1000) across three replicates.

	Concentration (µM)			
	**Trial 1**	**Trial 2**	**Trial 3**	**Average (±SD)**
**pX-6*6***	4	8	8	**8 (±2)**
**pX-P6*P6***	4	4	4	**4 (±0)**
**Mes-6*6*6***	4	4	4	**4 (±0)**
**Mes-P6*P6*P6***	8	8	16	**8 (±5)**
**1,4-6*6***	>250	>250	>250	**>250**
**1,4-P6*P6***	8	8	8	**8 (±0)**
**1,8-6*6***	16	16	16	**16 (±0)**
**1,8-P6*P6***	16	16	16	**16 (±0)**
**12(2)12**	16	16	16	**16 (±9)**
**MeP2P-12,12**	32	16	16	**16 (±9)**
**pX-12,12**	8	4	4	**4 (±2)**
**BEC**	63	32	32	**32 (±18)**
**DDAC**	32	16	16	**16 (±9)**

Table S8. Raw MBEC Data for CA-MRSA (USA300-0114) across three replicates.

	Concentration (µM)			
	**Trial 1**	**Trial 2**	**Trial 3**	**Average (±SD)**
**pX-6*6***	8	8	8	**8 (±0)**
**pX-P6*P6***	8	8	4	**8 (±2)**
**Mes-6*6*6***	8	16	8	**8 (±0)**
**Mes-P6*P6*P6***	16	16	16	**16 (±0)**
**1,4-6*6***	>250	>250	>250	**>250**
**1,4-P6*P6***	8	8	8	**8 (±0)**
**1,8-6*6***	63	63	32	**63 (±18)**
**1,8-P6*P6***	16	16	32	**16 (±9)**
**12(2)12**	63	63	32	**63 (±0)**
**MeP2P-12,12**	32	32	32	**32 (±0)**
**pX-12,12**	16	32	32	**32 (±9)**
**BEC**	125	63	63	**63 (±36)**
**DDAC**	16	32	32	**32 (±9)**

Table S9. Raw MBEC Data for HA-MRSA (ATCC 33591) across three replicates.

	**Concentration (µM)**			
	**Trial 1**	**Trial 2**	**Trial 3**	**Average (±SD)**
**pX-6*6***	>250	>250	>250	**>250**
**pX-P6*P6***	125	125	125	**125 (±0)**
**Mes-6*6*6***	125	125	125	**125 (±0)**
**Mes-P6*P6*P6***	63	63	63	**63 (±0)**
**1,4-6*6***	>250	>250	>250	**>250**
**1,4-P6*P6***	>250	>250	>250	**>250**
**1,8-6*6***	>250	>250	>250	**>250**
**1,8-P6*P6***	>250	>250	>250	**>250**
**12(2)12**	32	32	63	**32 (±18)**
**MeP2P-12,12**	32	32	16	**32 (±9)**
**pX-12,12**	32	32	32	**32 (±0)**
**BEC**	>250	>250	>250	**>250**
**DDAC**	250	250	125	**250 (±72)**

Table S10. Raw MBEC Data for *A. baumannii* (ATCC 17978) across three replicates.

	**Concentration (µM)**			
	**Trial 1**	**Trial 2**	**Trial 3**	**Average (±SD)**
**pX-6*6***	>250	>250	>250	**>250**
**pX-P6*P6***	>250	>250	>250	**>250**
**Mes-6*6*6***	>250	>250	>250	**>250**
**Mes-P6*P6*P6***	125	125	125	**125 (±0)**
**1,4-6*6***	>250	>250	>250	**>250**
**1,4-P6*P6***	>250	>250	>250	**>250**
**1,8-6*6***	>250	>250	>250	**>250**
**1,8-P6*P6***	250	250	250	**250 (±0)**
**12(2)12**	16	16	63	**16 (±27)**
**MeP2P-12,12**	16	16	32	**32 (±9)**
**pX-12,12**	16	16	8	**16 (±5)**
**BEC**	>250	>250	>250	**>250**
**DDAC**	>250	>250	>250	**>250**

Table S11. Raw MBEC Data for *P. aeruginosa* (PAO1) across three replicates.

	Concentration (µM)			
	**Trial 1**	**Trial 2**	**Trial 3**	**Average (±SD)**
**pX-6*6***	>250	>250	>250	**>250**
**pX-P6*P6***	>250	>250	>250	**>250**
**Mes-6*6*6***	>250	>250	>250	**>250**
**Mes-P6*P6*P6***	>250	>250	>250	**>250**
**1,4-6*6***	>250	>250	>250	**>250**
**1,4-P6*P6***	>250	>250	>250	**>250**
**1,8-6*6***	>250	>250	>250	**>250**
**1,8-P6*P6***	250	250	250	**250 (±0)**
**12(2)12**	125	125	125	**125 (±0)**
**MeP2P-12,12**	>250	>250	>250	**>250**
**pX-12,12**	63	125	125	**125 (±36)**
**BEC**	>250	>250	>250	**>250**
**DDAC**	>250	>250	>250	**>250**

2. Supporting Figures

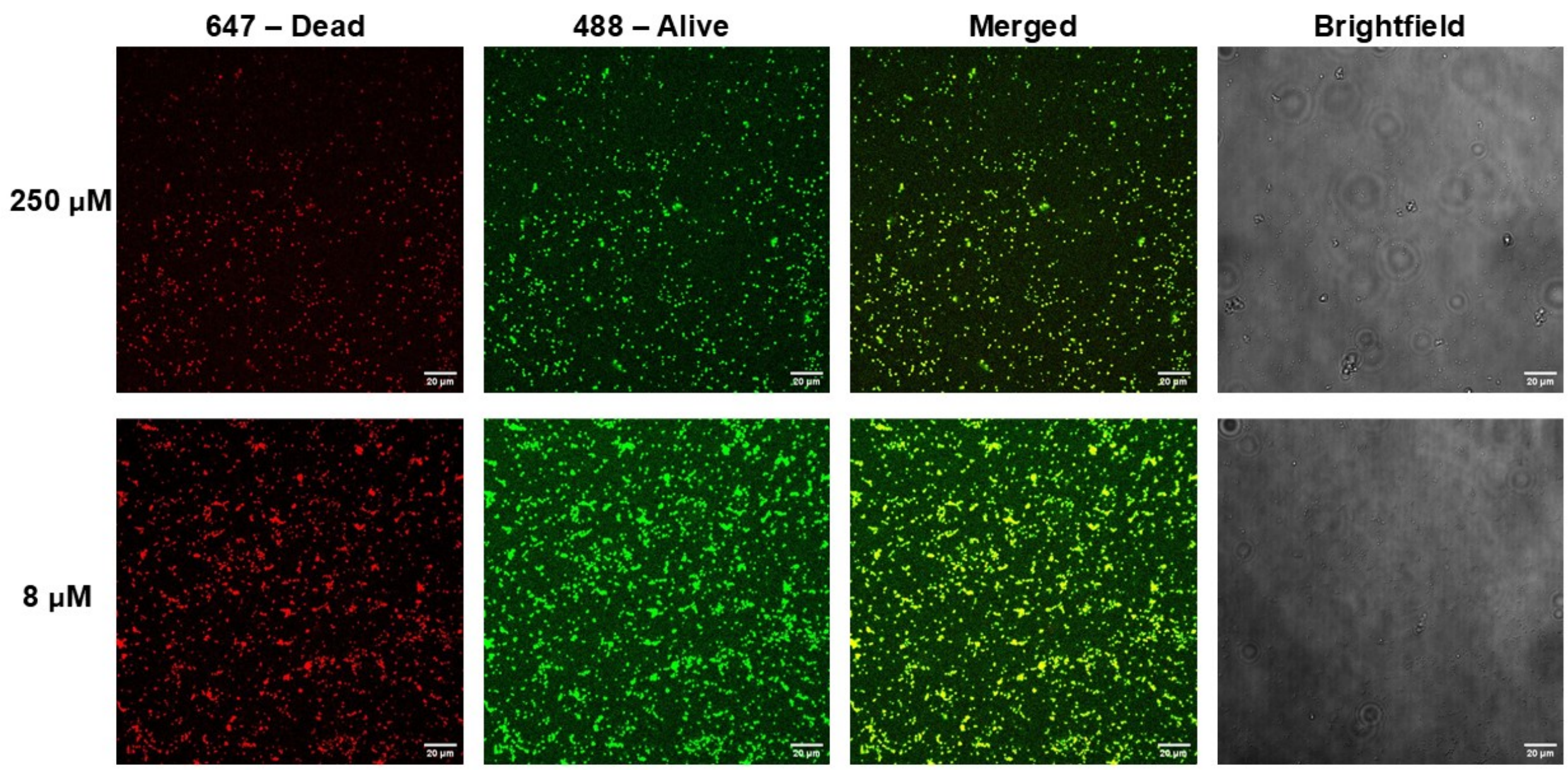

Figure S1. Individual channel images for MSSA (SH1000) biofilm response to treatment with **pX-6*6*** (MBEC = 8 µM). Confocal images were taken with a 60× objective and LIVE/DEAD stain was used to visualize cell viability. Images contain a 20 µm scale bar.

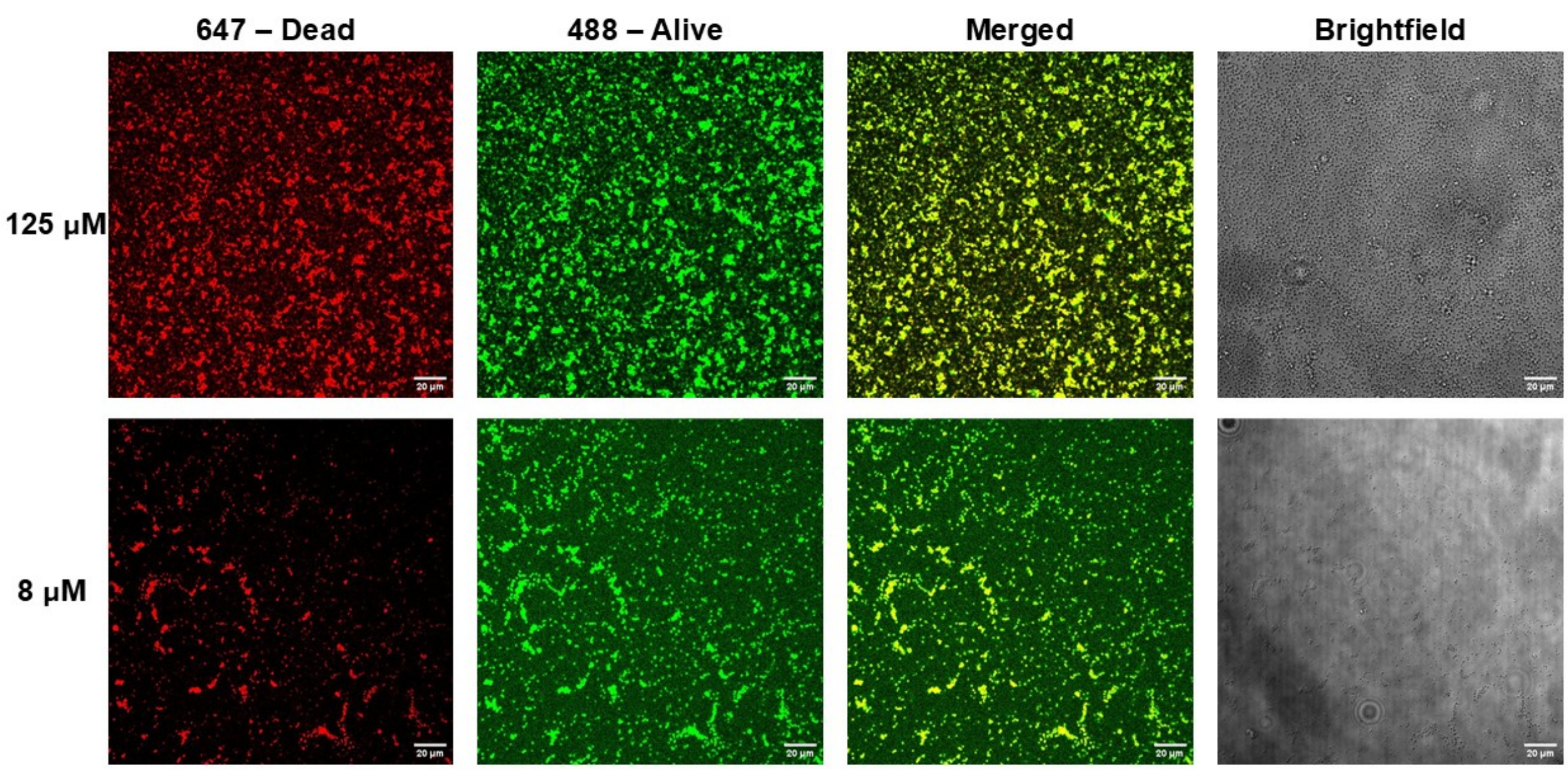

Figure S2. Individual channel images for. MSSA (SH1000) biofilm response to treatment with **pX-P6*P6*** (MBEC = 8 µM). Confocal images were taken with a 60× objective and LIVE/DEAD stain was used to visualize cell viability. Images contain a 20 µm scale bar.

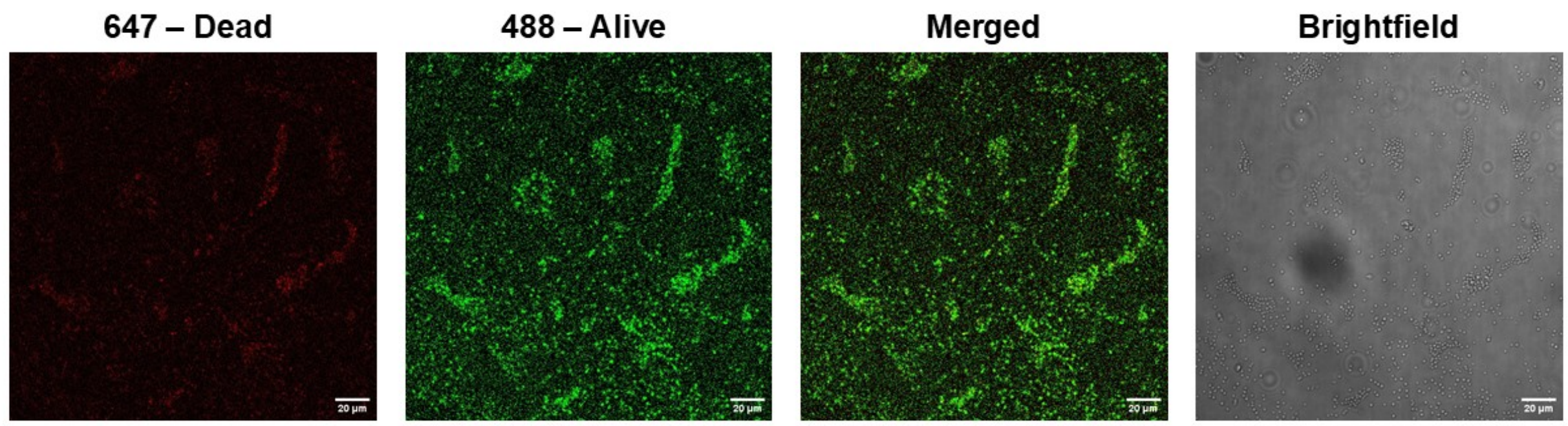

Figure S3. Individual channel images for untreated MSSA (SH1000) biofilm. Confocal images were taken with a 60× objective and LIVE/DEAD stain was used to visualize cell viability. Images contain a 20 µm scale bar.

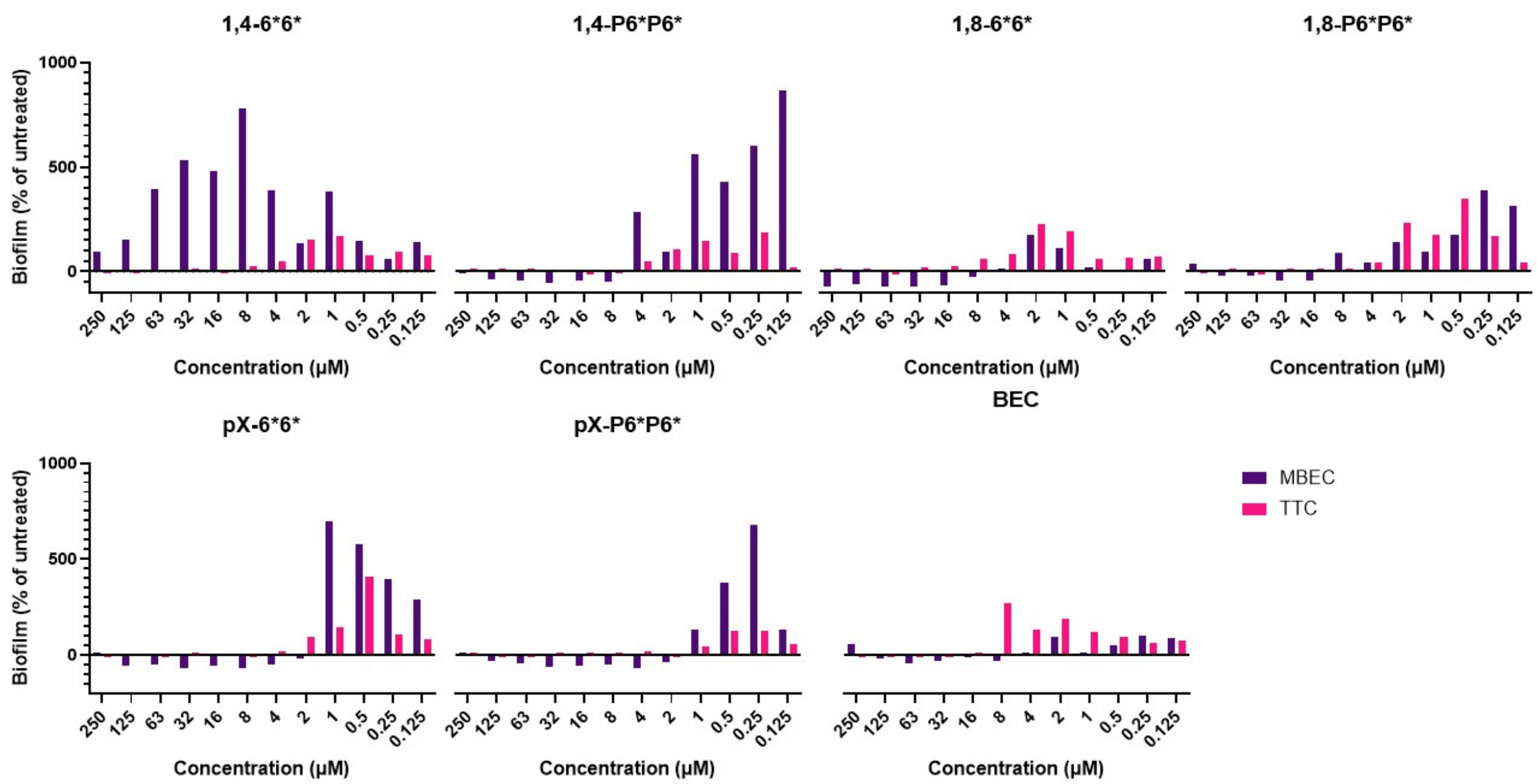

Figure S4. Quantification of crystal violet staining and metabolic activity using TTC dye to assess whether biofilms are metabolically active at high disinfectant concentration. Purple bars represent CV staining and pink bars represent TTC.

3. Spectra

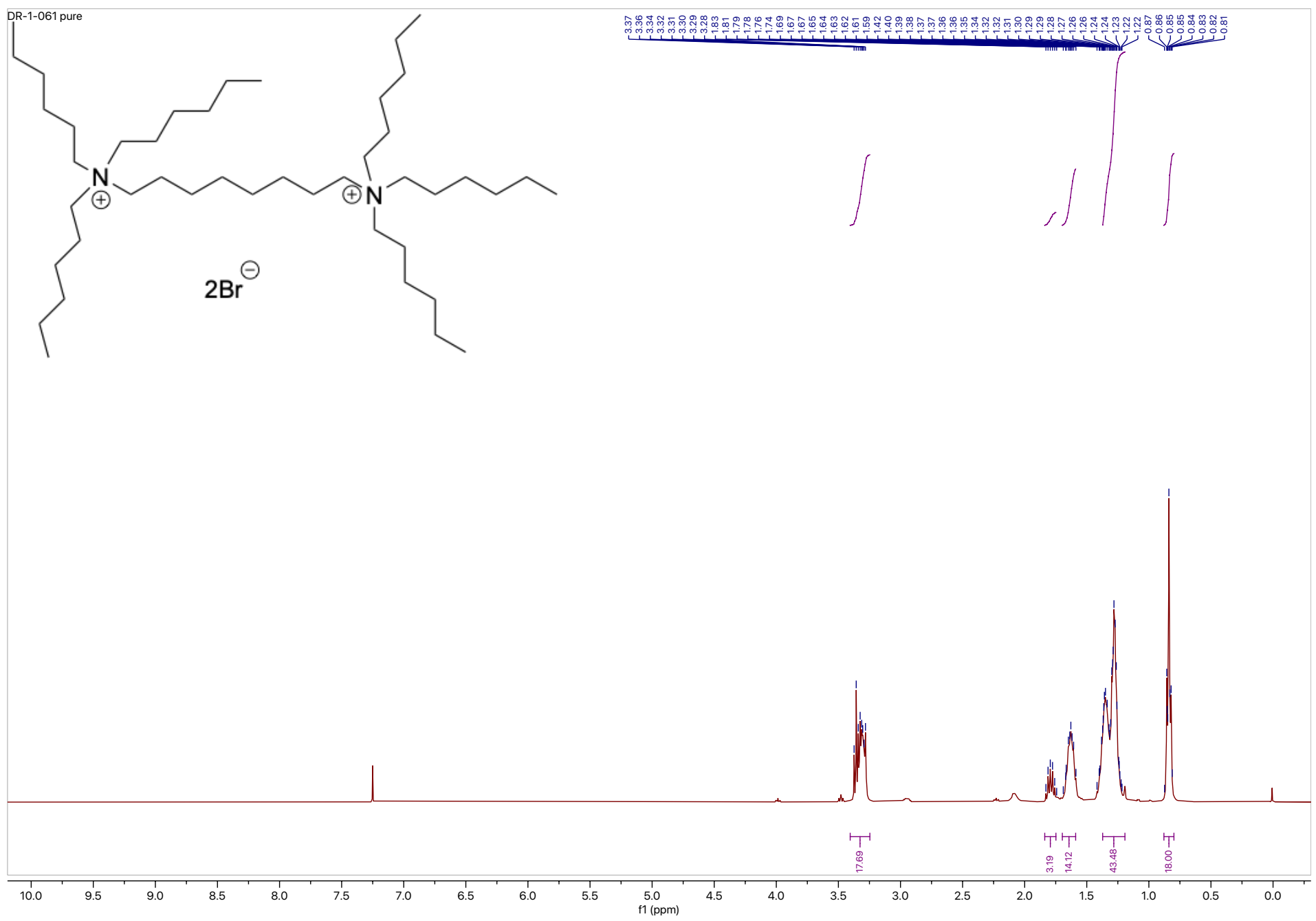

^{1}H NMR of **1,8-6*,6*** in $CDCl_3$

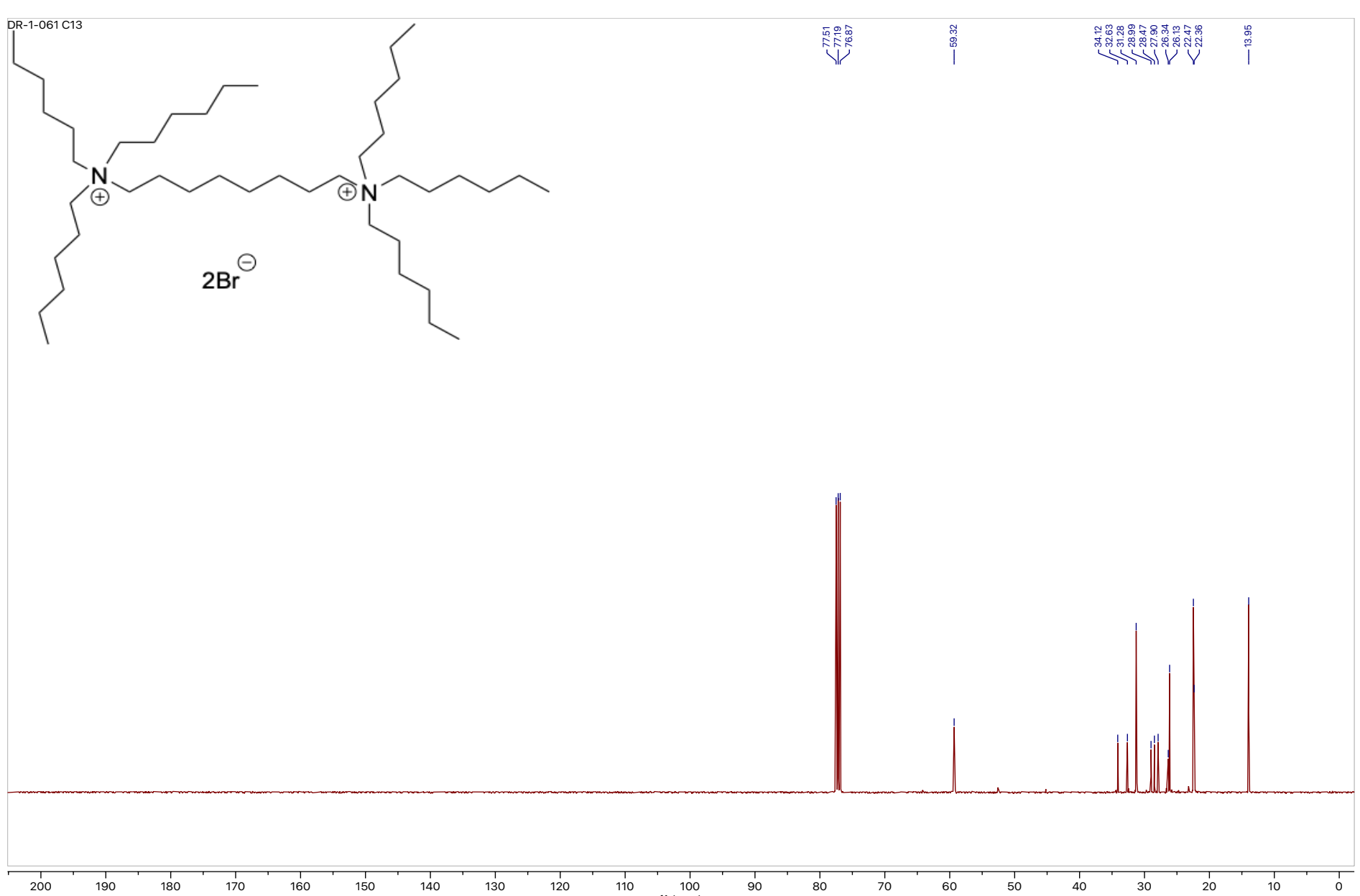

^{13}C NMR of **1,8-6*,6*** in $CDCl_3$
